# The Characterization and Stability of Powdered Oil Loaded with β-Carotene Prepared from a Sodium Caseinate–Carrageenan Complex: The Effect of Vacuum Freeze-Drying and Spray-Drying

**DOI:** 10.3390/foods13223690

**Published:** 2024-11-19

**Authors:** Yue Long, Juan Zhang, Delong Li, Yanpeng Zhang, Yang Cao, Wei Xu, Zhixiong Hu, Chun Hu

**Affiliations:** 1 College of Food Science and Engineering, Wuhan Polytechnic University, Wuhan 430023, China; yuelong0408@163.com (Y.L.); zhangjuanzj01@163.com (J.Z.); ldl7128@163.com (D.L.); xuwei1216@163.com (W.X.); e_huzhixiong@126.com (Z.H.); huchun202020@163.com (C.H.); 2 Key Laboratory for Deep Processing of Major Grain and Oil, Ministry of Education, Wuhan Polytechnic University, Wuhan 430023, China; 3 Hubei Key Laboratory for Processing and Transformation of Agricultural Products, Wuhan Polytechnic University, Wuhan 430023, China

**Keywords:** β-carotene, wall material, drying method, encapsulation, reconstitution

## Abstract

β-carotene (BC) has various biological activities, such as anticancer properties, contributing to the prevention of cardiovascular diseases, etc., while the poor solubility and low bioavailability limit its further development in the food industry. Therefore, how to effectively encapsulate this unstable substance has become a hot topic. Here, different concentrations of sodium caseinate and ι-carrageenan (NaCas-CA) complex emulsions were used as wall materials, and the effect of spray-drying (SD) and vacuum freeze-drying (VFD) on the properties of BC powders loaded with NaCas-CA was investigated. The results showed that the characteristic peaks of BC disappeared in all powdered oils, indicating that BC could be effectively encapsulated. As the wall concentration increased, the surface oil of the powders decreased significantly. At the same concentration, the surface oil content of SD (minimum of 8.34%) was lower than that of VFD (minimum of 10.02%). However, the particle size of SD-reconstituted emulsions was larger than that of VFD. Furthermore, the SD-reconstituted emulsions were more stable than the VFD after storage at 25 °C for 3 h. This study reveals the effect of different drying methods on the structure and stability of powdered oils, providing valuable information for the research of functional active-loaded powdered oils and applications in the food industry.

## 1. Introduction

β-carotene (BC) is an important nutrient supplement and active substance in food systems as a natural pigment, antioxidant, and efficient vitamin A precursor [1]. However, its poor water solubility and low bioavailability often limit its further application, making it urgent to maintain the stability of BC and construct a natural, green, and healthy food system. In recent years, a series of food delivery systems have been investigated to protect BC and improve its utilization, such as emulsions, micelles, liposomes, protein nanoparticles, hydrogel particles, and solid lipid nanoparticles, which can improve the bioavailability of hydrophobic nutrients or active substances and promote the physiological functions by increasing their water solubility, stability, and the absorption efficiency of water [2].

One of the strategies is the emulsion delivery system, which can be used to increase the dispersion of hydrophobic nutrients, prevent the oxidative degradation, and enhance the bioavailability of BC. Nie et al. [3] prepared high internal phase Pickering emulsions (HIPPE) of peanut isolate protein and cellulose nanocrystals for the encapsulation of BC, which demonstrates that the encapsulation BC in the emulsion not only improves its solubility, but also protects it from degradation by adverse factors in the external environment, thus increasing its bioavailability [4]. In practice, however, emulsions tend to settle and delaminate during long-term storage and are difficult to transport or use in some food systems as a result of gravity and molecular Brownian motion. To extend the shelf life of emulsion products, drying technology is used to convert the emulsion into powdered oil, where the wall material encapsulates the bioactive substance, providing a physical barrier that prevents molecular diffusion and chemical reactions, thus increasing the stability of the encapsulated compounds [5]. Over the years, many studies have been conducted on powdered oils for BC encapsulation. For example, Ashay et al. [6] used the isolated whey protein and gum arabic as biopolymers to protect BC and achieve controlled release. Another study also proved that the coacervates of pullulan polysaccharide and whey protein complex made for an excellent wall material for the encapsulation of BC [7]. These studies demonstrated that emulsions stabilized by protein–polysaccharide particles can become an effective delivery systems for hydrophobic bioactive compounds by drying.

Spray-drying (SD) and vacuum freeze-drying (VFD) are common drying methods for the preparation of powdered oil. SD is a drying method in which atomized materials are converted into powders in one step by evaporating water at high temperatures. Because of its flexibility and continuous production process, SD is considered a common industrial-scale drying technique [8]. Freitas et al. [9] successfully microencapsulated tucumã oil by spray-drying, spray-cooling and a combination of the two methods. However, both unencapsulated and encapsulated tucumã oils presented low BC bioaccessibility. VFD is a drying method that sublimates moisture in frozen materials under high vacuum conditions, which is friendly to heat-sensitive bioactives [10]. Since the drying process is carried out at low temperatures, the oxidation and chemical denaturation of heat-sensitive components of the product can be inhibited [11]. Lin et al. [12] prepared and lyophilised emulsions of BC-loaded soluble complexes formed from whey proteins and OSA-modified starch, and BC-loaded soluble complexes lyophilized had good reconstitution properties. The application and research of these technologies show that the efficient control of drying technology and the choice of wall materials are the key to the encapsulation of BC.

Sodium caseinate (NaCas) as an emulsifier is amphiphilic and highly surface-active, and it can adsorb on the surface of droplets to reduce the surface tension [13]. ι-carrageenan (CA), as an emulsion stabilizer, can improve the viscosity of the continuous phase and the strength of the interfacial film, providing an ideal emulsion texture [14]. In addition, there is excellent potential for the stability of loaded BC emulsions formed by aggregation interactions between protein–polysaccharide complexes [15]. It has been shown that NaCas and CA conjugates formed by glycation can significantly enhance the stability of HIPPE [16]. Therefore, NaCas-CA complexes can effectively improve the structure of the emulsion interface, increase the steric hindrance and electrostatic repulsion of the oil–water interface, and promote the stability and environmental adaptability of the emulsion systems [17]. These properties demonstrate their excellent potential as carrier matrices for the encapsulation and delivery of hydrophobic bioactive ingredients.

In addition, our research group has successfully prepared the emulsion system loaded with BC using NaCas-CA complexes, which showed satisfactory stability, pH-responsive release ability, and bioavailability [18]. The NaCas-CA complex-loaded BC achieved controlled and targeted release in the small intestine, with a bioavailability of 80.2%. Therefore, it is hypothesized that NaCas-CA complexes could be an ideal wall material for BC encapsulation. Here, the objective of this study is to determine the appropriate wall/core ratio and to investigate the influence of drying methods on the surface oil content, microstructure, and other properties of powdered oils, in order to provide a theoretical basis for the application of BC and other active substances in the food industry and the healthcare product industries.

## 2. Materials and Methods

### 2.1. Materials

CA and NaCas were purchased from Sigma-Aldrich Co., Ltd. (Shanghai, China). BC was provided by Shanghai Aladdin Biochemical Technology Co., Ltd. (Shanghai, China). Corn oil was obtained from Yihai Kerry Arawana Holdings Co., Ltd. (Wuhan, China). Other chemicals and reagents were analytical grade and commercially purchased from Sinopharm Chemical Reagent Co., Ltd. (Shanghai, China). Deionized water was used throughout all experiments.

### 2.2. Preparation and Characterization of Powdered Oils

#### 2.2.1. Preparation of the NaCas-CA Complex

According to our previous study [19] and the conditions determined by the preliminary experiment, the NaCas-CA complex solution was used as the wall material, and the wall-material concentrations in this investigation were selected as 1.3%, 1.5%, and 1.7% (*w*/*w*), respectively. Briefly, the solution of the NaCas-CA complex was prepared by mixing NaCas and CA in a 2:1 ratio (*w*/*w*) in a beaker and stirring at 400 rpm for 1 h on a magnetic stirrer (MS-M-S10, DLAB, Beijing, China), followed by heating in a water bath (DF-101 S, LICHEN, Shanghai, China) at 85 °C for 30 min. The pH was adjusted to 6.0 with 0.1 mol/L of HCl and then the solution was stirred homogeneously at 400 rpm with a magnetic stirrer at room temperature.

#### 2.2.2. Preparation of the NaCas-CA-Loaded BC Emulsions and Powdered Oils

The NaCas-CA-loaded BC emulsions were prepared according to the previously reported methods [19,20] with necessary modifications. The appropriate amount of BC was gradually added to the corn oil and stirred at 400 rpm for 1 h at 40 °C to reach a final concentration of 0.5% (*w*/*w*) in the oil phase. The prepared NaCas-CA complex emulsion was mixed with the oil phase in a 9:1 ratio (*w*/*w*) and homogenized at 10,000 rpm for 2 min using a homogenizer (T18 Ultra-Turrax, IKA, Staufen, Germany) to obtain the crude emulsion. Subsequently, the BC-loaded emulsion was prepared by homogenizing three times using a high-efficiency compressor (Nano DeBEE, BEE, South Easton, MA, USA) at 10,000 psi. The entire preparation process was carried out under dark protective conditions to reduce the loss of BC during the preparation process.

The powdered oils were obtained after SD and VFD treatment. For SD, samples were passed through a spray dryer (SP-1500, SUNYI, Shanghai, China) with a 0.7 mm two-fluid nozzle at a flow rate of 15 mL/min. At this point, the air inlet temperature was 160 °C and the air outlet temperature was 80 °C. For VFD, the samples were pre-freezed at −70 °C for 72 h in an ultra-low temperature freezer (DW-HL680, TUOXING, Hefei, China) and then freeze-dried using a lyophilizer (Alpha 1-2 LD plus, Christ, Osterode, Germany) for 72 h. The samples obtained were coded SD-1.3%, SD-1.5%, SD-1.7%, VFD-1.3%, VFD-1.5%, and VFD-1.7%, respectively.

#### 2.2.3. Surface Oil Content

The measurement of the oil content of the surface was performed according to a published method with some changes [21]. The Erlenmeyer flask was first dried to a constant weight, and 1 g of powdered oil was wrapped in absorbent cotton and placed in a funnel. Then, 20 mL of n-hexane was slowly poured into an absorbent cotton in the funnel, and the filtrate was collected in the constant-weight Erlenmeyer flask. The collected solvent was evaporated in a water bath and evaporated to a constant weight in an oven. The amount of oil on the surface was calculated using Equation (1):

(1) $$\mathrm{Surface}\;\mathrm{oil}\;\mathrm{content}\;(\%)=\frac{m_{2}-m_{1}}{m_{0}}\times 100$$

where *m*_0_ is the weight of the powdered oil, *m*_1_ is the weight of the Erlenmeyer flask, and *m*_2_ is the weight of the Erlenmeyer flask and residual oil after drying.

#### 2.2.4. Scanning Electron Microscopy (SEM)

A small amount of NaCas-CA-loaded BC powdered oil was fixed to the sample stage with a conductive carbon tape attached. The excess powder was gently blown off and coated with a thin layer of gold for 3 min. The microstructure of the powdered oils was observed by scanning electron microscope (SEM) (Regulus8100, Hitachi, Tokyo, Japan) at 2 and 3 kv with a magnification of ×100 and ×2000.

#### 2.2.5. Fourier Transform Infrared (FTIR)

The chemical structures of BC and powdered oils were characterized using a Fourier transform infrared spectrometer (Spectrum 100, PerkinElmer, Waltham, MA, USA) based on a previous method [22], and the air was used as a blank control. Briefly, 2.0 mg of the powdered oil was mixed with 198 mg of desiccated potassium bromide (KBr) and thoroughly ground. The mixture was compressed into a pellet and placed in the sample holder. The spectra were scanned from the 4000 to 250 cm^−1^ wavenumbers in 32 scans with a spectral resolution of 4 cm^−1^.

### 2.3. Preparation and Characterization of the Reconstituted Emulsion

#### 2.3.1. Reconstitution of Powdered Oils

The powdered oil sample of 1% (*w*/*w*) was dissolved in deionized water and stirred to homogeneity for 10 min at a temperature of 25 °C. It is necessary to note that the total solid content of the reconstituted emulsion should remain the same as that of the original NaCas-CA complex emulsions.

#### 2.3.2. Determination of the Particle Size and Zeta Potential

To avoid multiple scattering effects, the reconstituted emulsion was diluted 100 times with deionized water, and the particle size of the reconstituted emulsion was then measured with a laser particle-size analyzer (Mastersizer 3000, Malvern Instruments, Worcestershire, UK). The zeta potential was measured with a particle electrophoresis instrument (Zetasizer Nano ZS, Malvern Instruments, Worcestershire, UK). The original NaCas-CA complex emulsion was used as a control, and each group of samples was measured at a temperature of 25 °C in triplicate.

#### 2.3.3. Determination of the Stability of the Reconstituted Emulsion

The stability of the reconstituted emulsion was evaluated using a Turbiscan laboratory (Turbiscan Lab, Formulaction, Toulouse, France) based on the principle of multiple light scattering. The prepared reconstituted emulsion was immediately sampled, and 20 mL of the sample was transferred to the sample vial of the stability analyzer and measured at 25 °C. The sample was continuously scanned in multi-scan mode for 3 h to obtain a trend summary of multiple curves, and the TSI was analyzed by the TurbiSoft-lab software (Version 3.3.1) included with the stability analyzer.

#### 2.3.4. Analysis of BC Storage Stability

The powdered oils were stored in a refrigerator at 4 °C (BCD-336WDPC, Haier, Qingdao, China) and in a thermostatic storage cabinet at 25 °C (RSD-312W, RongShiDa, Hefei, China) in the dark, respectively. The retention rate of the BC was measured at different storage times according to the previously reported method [23]. A total of 1 mL of the sample was thoroughly mixed with 2 mL of ethanol and 3 mL of n-hexane, and shaken on a vortex instrument until completely dissolved. The mixture was then allowed to stand for a few minutes, and the extraction solution (n-hexane phase) was aspirated and collected. The above extraction process was repeated twice more, and the extraction solution was combined. After volume fixation, the extract was aspirated and the absorbance was determined using a UV–vis spectrophotometer (UV-6100, Metash, Shanghai, China) at a wavelength of 450 nm. The storage stability of pure BC powder was used as a control and the retention rate of BC during storage at different temperatures was calculated by Equation (2):

(2) $$\mathrm{Retention}\;\mathrm{rate}\;(\%)=\frac{C_{n}}{C_{0}}\times 100$$

where *C*_0_ and *C_n_* are the BC contents in the initial powdered oil and on day *n*.

### 2.4. Statistical Analysis

Each experiment was repeated in triplicate and the results were expressed as mean ± standard deviation. The Origin 2018 (OriginLab, Northampton, MA, USA) was used for plotting, and significance analysis (*p* < 0.05) was performed using SPSS software (SPSS 19.0, IBM, Chicago, IL, USA).

## 3. Results

### 3.1. Characterization of Powdered Oils

#### 3.1.1. Surface Oil Content of Powdered Oils

**Figure 1:**
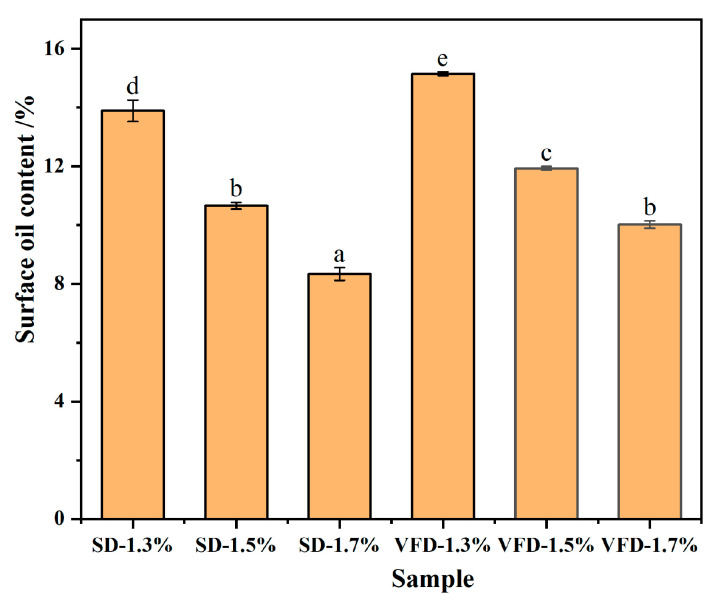
Surface oil content of different powdered oils. Different lowercase letters indicate significant differences (*p* < 0.05) among treatments.

Surface oil measurement is a method of evaluating the efficiency of encapsulation by determining the amount of oil that remains unencapsulated in the wall material and exposed to the air. The reduction in surface oil indicates an improvement in encapsulation efficiency, since it is impossible to reduce surface oil merely by controlling the parameters of the drying equipment [24]. Therefore, it can be controlled by changing the ratio of the core to the wall of the emulsion before drying or by changing the drying method [25]. As shown in Figure 1, the surface oil content of both drying methods decreased with an increasing concentration of wall material. The reason for this was that, when the content of the wall material was relatively low, the oil would not be completely encapsulated. When the concentration of the wall material increased, the formation rate and thickness of the protective layer around the oil increased, reducing the emission of oil content from the surface [8]. However, if the concentration of wall material was too high, it was too viscous to be fed to the homogenizer and was unsuitable for spraying, resulting in a dramatic reduction in encapsulation efficiency. The surface oil content of SD powder was lower than that of VFD powder (see Figure 1). Similarly, in the research project of Wang et al. [26], when an equal portion of maltodextrin, NaCas, and other additives were used to prepare microcapsules of walnut oil by SD and VFD methods, the surface oil residue of the powder prepared by SD was significantly less than that of VFD. Therefore, during the SD process, the synergistic effect between NaCas and CA could form the compact network structure and enhanced the comprehensive adsorption effect at the oil–air interface [27]. This effect reduced the migration of the core material (BC) to the surface of the powdered oil, thereby inhibiting the movement and agglomeration of the droplets. In contrast, the surface oil content of the VFD powders was relatively high. It was speculated that the emulsion would form ice crystals during pre-cooling, which destroyed the interfacial film of the emulsion. Then, in VFD, the ice crystals sublimate and escape, forming a sponge-like microstructure, thus increasing the surface oil content of VFD powders [28]. This is the reason why the powders obtained by SD were pale yellow, while the powders obtained by VFD were darker.

#### 3.1.2. Microscopic Morphology of Powdered Oils

**Figure 2:**
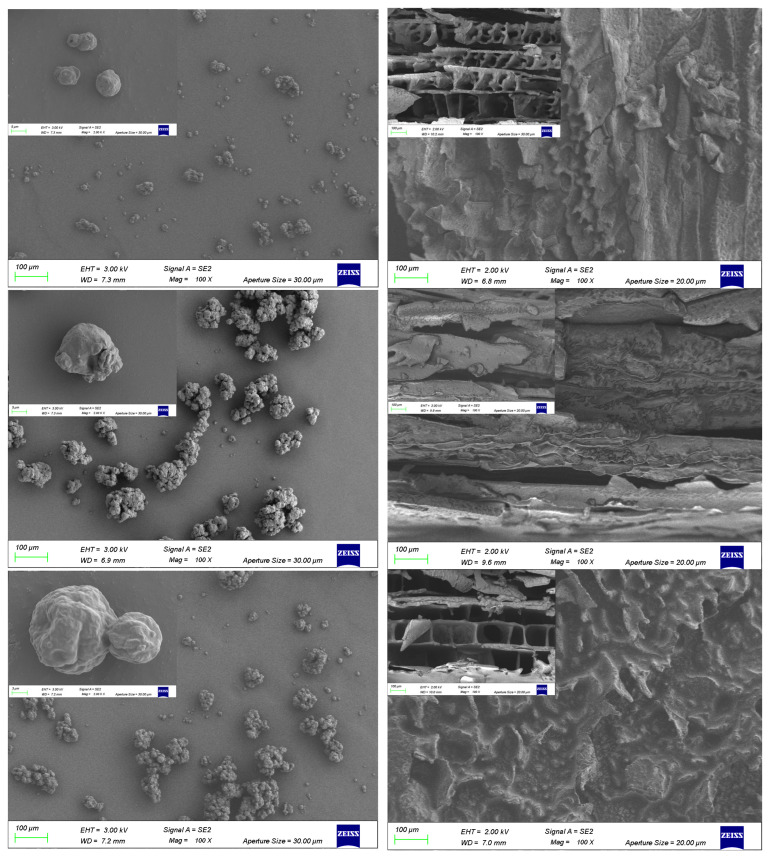
SEM of BC powdered oils produced by the two processes at 2 and 3 kV with magnifications of ×100 and ×2000. (From top to bottom on the left: SD-1.3%, SD-1.5%, SD-1.7%; from top to bottom on the right: VFD-1.3%, VFD-1.5%, VFD-1.7%).

The morphological structure of the powdered oils was further observed by SEM. As revealed in Figure 2, the morphological characteristics of the microparticles produced by the two drying methods were far different. The shape characteristic of SD powders at all wall concentrations was close to spherical or irregular, which was determined by the choice of wall material [29]. Although there were no visible cracks and/or fissures in the SD powders, the surface of some of the particles showed concave and shriveled shapes, which formed when the water in the emulsion evaporated rapidly, resulting in a sharp increase in surface tension [30]. Another possible explanation was that the structure of the network formed by NaCas-CA was destroyed by heat flow during SD, resulting in the formation of partial bubbles due to the low rigidity of the wall material, and the surface shrinkage was caused by the difference in pressure difference during cooling [31]. Furthermore, at a wall-material concentration of 1.3%, a large amount of sample was adsorbed on the wall of the atomization chamber, resulting in a low yield of powdered oil with a small particle size. Considering that higher concentrations of wall material would increase the viscosity of the system, larger droplets and particle sizes of powder could be formed in the atomization chamber, which was consistent with the results of the trehalose-corn oil nanoemulsion powder prepared and observed by Teo et al. [32]. At a wall-material concentration of 1.3%, the SEM micrograph revealed that some of the small particles were adsorbed onto the large ones. Not only that, some of the particles formed agglomerates as the wall-material concentration increased, but since individual particles were observed, it was demonstrated that the mutual collisions between the particles during the drying process facilitated adsorption and agglomeration [33]. This agglomeration could better protect the core material by reducing the contact surface of the powder with the air. Furthermore, most of the VFD powders showed a lamellar shape. Ribeiro et al. [34] found that the powder of roasted coffee oil had varying degrees of denting and breakage. A similar structure was also observed in our VFD-prepared NaCas-CA-loaded BC powders. These results were reasonable because of the aggregation of NaCas and CA by intermolecular forces to form a lamellar structure at low temperatures under the influence of intermolecular forces and the formation of ice crystals by the emulsions during freezing, which punctured the surface structure and formed many voids. Furthermore, as the concentration of wall material increased, the lamellar structure became more pronounced and was even destroyed into small fragments. The high brittleness of the lamellar structure was more easily destroyed, and the formation of small fragments increased the contact surface between the core material and the air [35]. In conclusion, morphological characterization demonstrated that VFD powders were unfavorable for BC encapsulation and stability compared to SD powders.

#### 3.1.3. FTIR Spectroscopy

**Figure 3:**
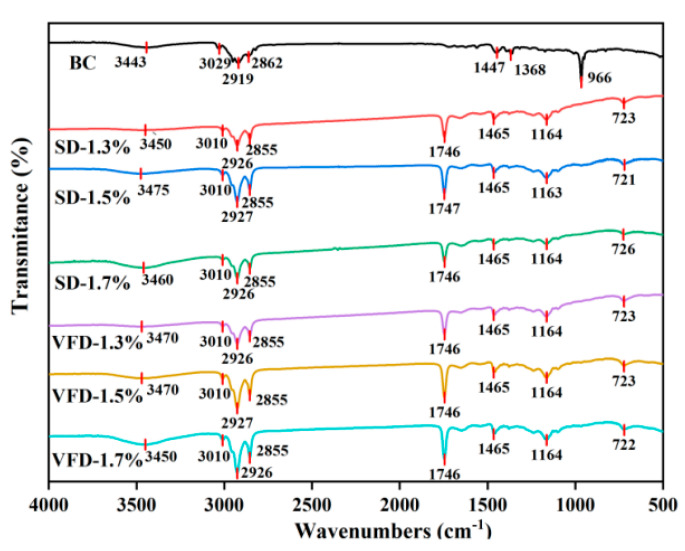
FTIR spectroscopy of BC and powdered oils treated by different treatments.

Figure 3 shows the FTIR profiles for pure BC and powdered oils with different treatments. The characteristic peaks of the infrared spectra of BC powdered oils obtained by SD and VFD were almost the same, indicating that the drying methods had a limited influence on the chemical bonding of the substances.

In the six sets of infrared spectra of powdered oils with different drying methods and wall concentrations, the weak and flat band around 3650−3200 cm^−1^ (−OH stretching) appeared in powdered oils under two drying methods, which was attributed to hydrogen bonds between molecules (NaCas and CA) or interactions with water. Therefore, the wall material acted as a protective agent for BC, and the higher the concentration of the wall materials, the higher the peak intensity, indicating a stronger protective effect. The weak peaks observed in the range of 1500−1100 cm^−1^ represented the bending vibration of methylene and the stretching vibration of CO. In particular, the peaks observed at 3000−2800 cm^−1^ in the triple bands (−CH stretching vibration), 1746 cm^−1^ (C=O stretching vibration) and 724 cm^−1^ (−CH deformation vibration) are the characteristic peaks of surface oil (corn oil) [36]. From the triple peaks and the C=O stretching vibration peaks, the peak intensity of SD relative to VFD was significantly lower, which was indicative of a lower surface oil content. Furthermore, as the concentration of the wall material increased, the peak intensity became weaker, indicating that the surface oil of the powdered oil decreased with the concentration of the wall material. Furthermore, from the BC FTIR spectrum of BC, 3443 cm^−1^ (O-H stretching), 3029 cm^−1^ (=CH stretching vibration), 2919 cm^−1^ (asymmetric stretching vibration of −CH), 2862 cm^−1^ (symmetrical stretching vibration of −CH), 966 cm^−1^ (R_1_HC=CHR_2_ wagging vibration) were the hallmark features of BC, as detailed by Reksamunandar et al. [37]. In the FTIR spectra of the powdered oil formed by the two drying methods, the characteristic peaks of BC were barely observed, which is consistent with the results of Zhang et al. [6], suggesting that BC was effectively encapsulated.

### 3.2. Characterization of Reconstituted Emulsions

The reconstitution capacity is considered as a critical quality benchmark for the consumption of powdered oils. The recombination behavior of the powder oil was investigated by measuring the particle size, zeta potential, and stability.

#### 3.2.1. Particle Size and Zeta Potential

The ionic nature of the rehydrated emulsion, which is crucial for storage stability, is significantly modulated by the status of the powdered oils, underscoring the effect of different drying methods on encapsulation and reconstitution. In general, emulsions with absolute values of zeta potentials within 20 mV are dominated by the attraction and aggregation of emulsion droplets, resulting in poor stability. When the absolute value of the zeta potential is greater than 20 mV, the repulsion between the particles exceeds the attraction force, resulting in a relatively stable system [38]. As can be seen in Table 1, the zeta potential of the samples was greater than −45 mV, suggesting the superior stability of the reconstituted emulsion. Compared to the SD and VFD treatments, the potential of the original emulsion with a wall concentration of 1.3% decreased from −49.03 ± 0.42 mV to −47.27 ± 0.63 and −47.80 ± 0.41, suggesting a weak electrostatic interaction that was unfavorable for agglomeration, which was consistent with our SEM results (Figure 2). Since negatively charged particles were relatively high, at a wall concentration of 1.5% after SD and VFD treatment (−52.23 ± 0.69 and −51.27 ± 0.54), it was speculated that the repulsive potential energy between the emulsion reconstitution droplets was weakened at a moderate concentration, which facilitated particle aggregation and the formation of a three-dimensional network structure on the surface of the droplets [39]. These effects could inhibit instability factors during emulsion storage, such as Ostwald ripening, coalescence, etc., and facilitate long-term stability after reconstitution.

Particle size is an important indicator of the stability of an emulsion system, and the larger the particle size, the more the emulsion system tends to precipitate, resulting in an inhomogeneous system. Compared to the original emulsion, the particle size of the reconstituted emulsions obtained by the two drying methods increased significantly (*p* < 0.05), while the zeta potential remained almost unchanged. This suggested that there was no significant change in the interaction force between NaCas and CA under the two drying methods, but the drying process has a certain degree of negative effect on the encapsulation effect of NaCas-CA. Therefore, the increased particle size could be related to a lack of homogenization and poor dispersion of the emulsions during the reconstitution process. In addition, particle size was negatively correlated with wall-material concentration, which meant that the low concentration of the wall material increased the surface oil content, which promoted agglomeration between particles, leading to the incomplete encapsulation and coalescence of emulsion droplets. Interestingly, compared to the SD powders, the VFD powders had a smaller particle size after reconstitution. This difference was attributed to the porosity of the VFD powders, which allowed water to easily penetrate the powder, improving its solubility and dispersibility [40].

#### 3.2.2. Stability of Reconstituted Emulsions

**Figure 4:**
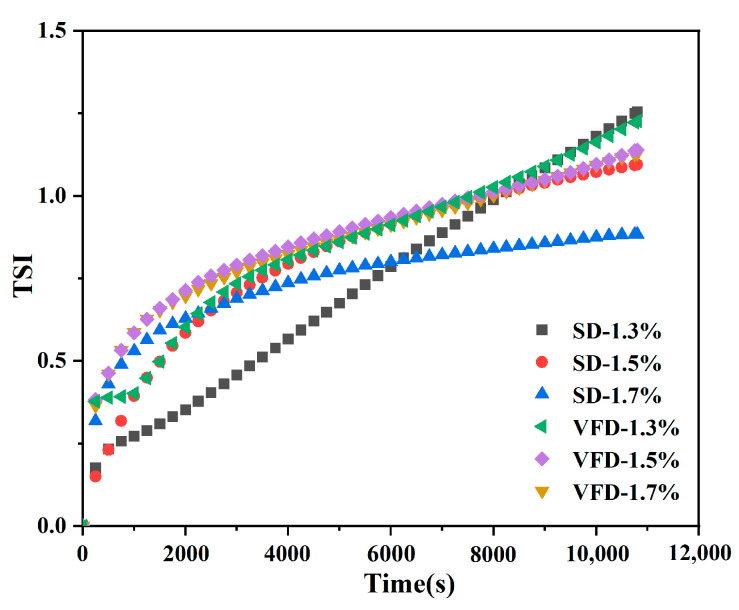
The total Turbiscan Stability Index (TSI) values of the reconstituted emulsions.

The Turbiscan Stability Index (TSI) was determined according to the variation in backscattered light, which is negatively correlated with the ability of powdered oils to form stable emulsions after reconstitution [23]. As shown in Figure 4, the dynamic instability of the reconstituted emulsions gradually increased with time due to the rapid aggregation of oil droplets under gravitational motion. The order of increase in TSI values after 3 h at 25 °C was SD-1.7% < SD-1.5% < VFD-1.7% < VFD-1.5% < VFD-1.3% < SD-1.3%. Both reconstituted emulsions at a wall-material concentration of 1.3% were unstable, indicating that emulsion droplets aggregated and settled faster at a low wall-material concentration, which was consistent with the observation of zeta potential and particle size (Table 1). Furthermore, the explanation for the increased stability of the reconstituted emulsions with increasing high-wall materials was that the formation of a dense interfacial layer suppressed the flocculation or agglomeration of oil droplets. The results of the experiment proved again that the SD method significantly improved the stability properties of the powdered oil compared to those of the VFD. It could be inferred that the formation of ice crystals during the VFD process punctured the surface film, resulting in a looser structure, and the core material (BC) was released and deteriorated during the reconstitution process, which was consistent with the SEM observations. However, the particle size of SD was larger than that of VFD (Table 1), suggesting that the small particle size of the emulsion was not necessarily a prerequisite for ideal stability. Instead, a small particle size implied that the oil droplets had a larger surface area, leading to a greater potential for oxidation [41].

#### 3.2.3. Storage Stability of BC

**Figure 5:**
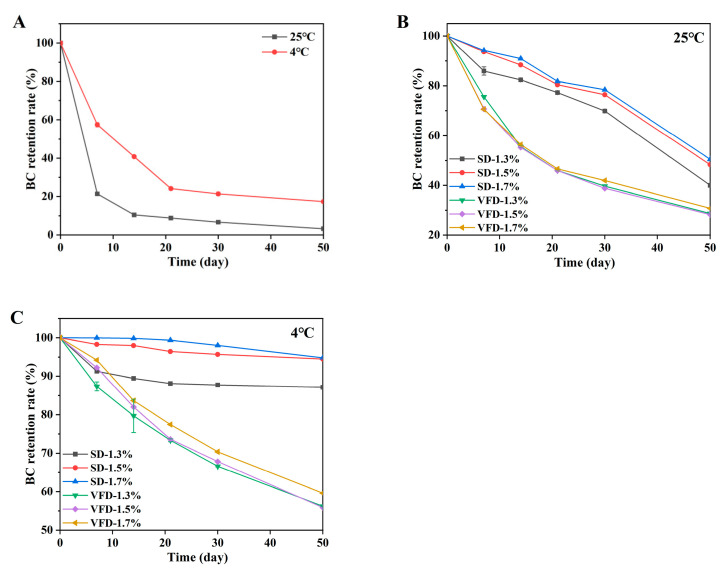
Retention of BC during storage at 25 °C and 4 °C for 50 days ((**A**): pure BC, (**B**): 25 °C, (**C**): 4 °C).

The storage stability of BC-loaded powders with two different drying treatments was evaluated when stored at the temperatures of 4 °C and 25 °C in the dark, respectively, and the results are shown in Figure 5. The pure BC retention rates at a temperature of 25 °C and 4 °C at day 50 were 3.21 ± 0.05% and 17.33 ± 0.50%, respectively. In general, BC retention values under two different drying methods were higher than pure BC powder (Figure 5A) at all temperatures. At 4 °C, the retention of SD-1.5% and SD-1.7% BC increased dramatically to 94.49 ± 0.10% and 94.8 ± 0.19% at the end of 50 days of storage. Meanwhile, the retention rates of SD-1.5% and SD-1.7% at 25 °C were 48.31 ± 0.10% and 50.45 ± 0.14% (Figure 5B), almost half the retention rate than at 4 °C. These results suggested that the storage temperature had a significant effect on the stability and degradation of BC, which was related to the physical and chemical properties of BC, and was in good agreement with the previous study in which BC was encapsulated by carboxylic curdlan conjugates grafted with ferulic acid, and the storage stability decreased with increasing temperature [42]. These results suggested that high temperatures tend to lead to the degradation of BC during storage. The diffusion of oxygen at the preserved emulsion temperature of 4 °C was reduced compared to 25 °C, thus preventing the oxidation of BC [43]. In addition, the results suggested that, compared with the VFD method, the SD method had a better protective effect on BC against chemical degradation in the emulsions at common storage temperatures, which was supported by the results of the stability studies of powdered oils and reconstituted emulsions. Furthermore, the higher the concentration of wall material, the higher the retention rate. This was because the higher the proportion of wall materials that can form a more compact network structure, the better the effect of encapsulation of internal active substances. However, when the wall concentration was 1.5% and 1.7%, there was no clear difference in the BC retention rate. In the same mass, the higher the percentage of wall material, the lower the total amount of relatively encapsulated active substance. For economic benefits, SD-1.5% is more economical in industrial production.

## 4. Conclusions

This work developed a NaCas-CA complex emulsion at different concentrations and explored its potential application as a wall material for β-carotene encapsulation. The FTIR results showed that the hydrogen bonds between NaCas and CA molecules or the interaction with water promoted the efficient encapsulation of BC. Furthermore, the microstructure revealed that the SD-prepared powdered oils formed a more compact spherical structure under electrostatic interaction. In contrast, VFD-prepared powdered oil, although capable of encapsulating BC, was less stable due to the formation of a loose and hollow network structure. Notably, the concentration of the wall material was negatively correlated with the surface oil content, as evidenced by the characteristic FTIR peaks around the surface oil. After reconstitution, VFD emulsion has smaller particle size and inferior storage stability than that of SD due to the loose and porous structure. BC storage stability analysis confirmed that powdered oil prepared under optimal processing conditions showed a preservation rate of more than 90% after 50 days of storage at 4 °C. This study was the first to evaluate the effects of SD, VFD, and wall-material concentration on NaCas-CA-loaded BC powders and reconstituted emulsions. The experimental results from the microstructure of the powdered oils and the stability of the reconstituted emulsions suggest that further study of the differences between the reconstituted emulsion and the original emulsion is necessary to understand the effect of drying on the encapsulation effect of NaCas-CA. This work provides a preliminary study of the selection of wall materials and drying methods for powdered oils loaded with BC and other bioactive compounds, which provides a valuable reference for its food processing application as fat supplements, flavorings, or food processing for milk powders, and various functional solid beverages.

## Figures and Tables

**Table 1:** Particle size and zeta potential of the original and reconstituted emulsions.

Sample	Particle Size	Zeta Potential
	(μm)	(mV)
Original emulsion-1.3%	0.440 ± 0.005 ^f^	−49.03 ± 0.42 ^abc^
Original emulsion-1.5%	0.357 ± 0.001 ^g^	−52.00 ± 0.99 ^e^
Original emulsion-1.7%	0.328 ± 0.015 ^g^	−50.07 ± 0.66 ^cd^
SD-1.3%	2.408 ± 0.029 ^a^	−47.27 ± 0.63 ^a^
SD-1.5%	2.203 ± 0.009 ^b^	−52.23 ± 0.69 ^e^
SD-1.7%	2.227 ± 0.012 ^b^	−48.97 ± 0.95 ^bc^
VFD-1.3%	1.940 ± 0.064 ^c^	−47.80 ± 0.41 ^ab^
VFD-1.5%	1.773 ± 0 012 ^d^	−51.27 ± 0.54 ^de^
VFD-1.7%	1.677 ± 0 017 ^e^	−50.83 ± 0.39 ^de^

Note: Values of the same columns without the same letter (s) indicate that they were significantly different in *p* < 0.05.

## Data Availability

The original contributions presented in the study are included in the article, further inquiries can be directed to the corresponding authors.
